# Patterns of discussion on neuroticism and self-management behaviors in type 2 diabetes: a scoping review using machine learning-assisted text mining

**DOI:** 10.3389/fpubh.2025.1708967

**Published:** 2025-11-04

**Authors:** Wei Sun, Dong Xie, Menglin Zhang, Wenhui Hou, Ziru Wang, Chendi Wang, Guodong Xu, He Yang

**Affiliations:** School of Nursing, Changchun University of Chinese Medicine, Jilin, China

**Keywords:** neuroticism, type 2 diabetes mellitus, self-management behaviors, machine learning, text mining

## Abstract

**Background:**

Self-management behaviors, including diet control, medication adherence, blood glucose monitoring, and physical activity, are crucial for type 2 diabetes management. Neuroticism, a personality trait associated with anxiety and stress sensitivity, may significantly influence these behaviors. However, a comprehensive synthesis of evidence is lacking.

**Objective:**

This scoping review aims to systematically map and synthesize how neuroticism has been examined in relation to self-management behaviors among adults with type 2 diabetes, and to identify recurring thematic patterns and knowledge gaps through machine learning–assisted text mining.

**Methods:**

A scoping review was conducted in PubMed, Scopus, Web of Science, Embase, CINAHL, PsycINFO, and the Cochrane Library, covering the period from database inception to September 2025. The search strategy included keywords such as “neuroticism,” “personality traits,” “type 2 diabetes,” “self-management,” and “adherence.” We used machine learning–assisted literature mining to summarize thematic patterns across included studies. The study selection process and workflow were conducted in accordance with the PRISMA-ScR guidelines.

**Results:**

Ten studies were included. Across the literature, neuroticism was most frequently discussed alongside blood glucose monitoring, followed by diet control, medication taking, and exercise. Psychological constructs such as anxiety, stress sensitivity, and social support were commonly co-mentioned in these discussions. Machine learning–assisted analyses highlighted recurring topics, concept clusters, and co-occurrence patterns that characterize the discourse on neuroticism and T2DM self-management.

**Conclusion:**

This scoping review characterizes how neuroticism is positioned within the discourse on T2DM self-management behaviors and delineates prominent thematic linkages and gaps. Machine learning–assisted text mining proved useful for organizing and visualizing dispersed evidence. Findings describe patterns in the literature rather than estimating causal effects, and can inform future hypothesis-driven studies and tailored clinical inquiry.

**Systematic review registration:**

Unique Identifier: 10.17605/OSF.IO/54NJD; publicly accessible URL: https://doi.org/10.17605/OSF.IO/54NJD.

## 1 Introduction

Type 2 diabetes mellitus (T2DM) is a chronic metabolic disorder characterized by insulin resistance and relative insulin deficiency, with an insidious onset, a prolonged course, and a high risk of complications (1–3). According to the International Diabetes Federation, approximately 537 million adults worldwide were living with diabetes in 2021, a number projected to increase to 643 million by 2030 (4). T2DM poses significant physical and psychological challenges for patients and imposes a heavy burden on global health systems and socioeconomic development (5). Effective management of T2DM requires sustained self-management behaviors beyond pharmacological treatment, including dietary regulation, regular physical activity, medication adherence, and blood glucose monitoring (6–8). These behaviors are fundamental to maintaining glycemic control, reducing complications, and improving quality of life. However, adherence to self-management practices remains suboptimal in clinical settings, suggesting that psychosocial and individual-level determinants warrant further exploration. Recent evidence highlights the growing importance of personality traits in shaping chronic disease management (9). Within the five-factor model of personality, neuroticism—defined as a tendency toward emotional instability, anxiety, and heightened stress reactivity—has attracted increasing attention for its potential relationship with health-related behaviors (10, 11). In patients with T2DM, higher neuroticism scores have been linked to poorer dietary regulation, irregular physical activity, inconsistent medication use, and lower frequency of glucose monitoring (12–14). Nevertheless, existing studies exhibit notable heterogeneity in design, measurement tools, and conceptual framing. The literature is fragmented and often descriptive, making it difficult to discern how the relationship between neuroticism and self-management behaviors has been examined and conceptualized across different studies. Given these inconsistencies, a scoping review is warranted to systematically map and synthesize how neuroticism has been studied in relation to self-management among adults with T2DM. Unlike systematic reviews that seek to quantify effect sizes or evaluate evidence quality, a scoping review aims to identify the breadth, scope, and conceptual patterns of existing research. This approach allows for the integration of diverse methodologies and provides a descriptive overview of emerging themes and research gaps.

To enhance transparency and analytical depth, this review incorporates machine learning–assisted text mining to identify recurring topics, co-occurrence patterns, and thematic linkages within the existing literature. By visualizing how neuroticism is positioned within discussions of T2DM self-management, this review seeks to establish a structured knowledge framework that highlights areas of convergence and fragmentation, thereby informing future hypothesis-driven research and tailored clinical strategies.

## 2 Methods

### 2.1 Study design

This study was conducted as a scoping review and was designed and reported in accordance with the PRISMA-ScR guidelines (15). The protocol was registered with the Open Science Framework^1^ and is publicly accessible. Consistent with its scoping review methodology, the study aimed to map the breadth and conceptual structure of existing evidence rather than quantitatively synthesize effect sizes or assess the quality of individual studies.

### 2.2 Research questions

An initial exploratory search of the literature was undertaken to refine the research question and clarify the scope of inquiry. The primary objective of this review was to map and synthesize how neuroticism has been examined in relation to self-management behaviors among adults with type 2 diabetes mellitus (T2DM). Based on this preliminary search, the following objectives were established: (1) Identify existing studies addressing neuroticism within the context of T2DM self-management; (2) summarize how neuroticism has been situated across key domains of self-management, including dietary control, physical activity, blood glucose monitoring, and medication adherence; (3) and delineate recurring thematic patterns, conceptual linkages, and research gaps in the current evidence base. Accordingly, the overarching question guiding this review was as follows: “How has neuroticism been examined and discussed in relation to self-management behaviors among adults with type 2 diabetes mellitus?”

### 2.3 Search strategy

Following the PRISMA-ScR reporting guidelines and the Arksey and O’Malley framework (15, 16), a comprehensive search was conducted to identify studies examining the relationship between neuroticism and self-management behaviors among patients with type 2 diabetes mellitus (T2DM). The databases searched included PubMed, Scopus, Web of Science, Embase, CINAHL, PsycINFO, and the Cochrane Library, covering all records from database inception to 8 September 2025. The search strategy combined controlled vocabulary (MeSH terms) and free-text keywords, including “neuroticism,” “personality traits,” “self-management,” “type 2 diabetes mellitus,” “dietary control,” “physical activity,” “blood glucose monitoring,” and “medication adherence.” Boolean operators (AND, OR), truncation symbols (“”*), and field tags [Title/Abstract] were applied to ensure a comprehensive search. The complete PubMed search syntax is presented in Supplementary material S1. In addition, the reference lists of included studies and relevant reviews were manually screened to ensure comprehensive coverage of eligible literature.

### 2.4 Eligibility criteria

#### 2.4.1 Inclusion criteria

Inclusion criteria included studies that include patients diagnosed with type 2 diabetes mellitus (T2DM) as the study population; studies examining neuroticism and its influence on self-management behaviors, including dietary control, physical activity, blood glucose monitoring, and medication adherence; original quantitative research (cross-sectional, cohort, or interventional designs) or mixed-method studies; publications available in English or Chinese; and studies providing full-text access with sufficient data for analysis.

#### 2.4.2 Exclusion criteria

Exclusion criteria included studies involving animals, case reports, reviews, or commentaries; studies that did not examine neuroticism or self-management behaviors; duplicate publications, with the most recent or most complete data retained; studies lacking full-text access or with substantial missing data; and studies that did not include patients with type 2 diabetes mellitus (T2DM).

### 2.5 Study selection

All the retrieved records were imported into EndNote 20 for de-duplication. Two independent reviewers screened titles and abstracts according to predefined inclusion and exclusion criteria. Before formal screening, both reviewers underwent calibration training and pilot testing to ensure consistency in the application of the criteria. Inter-rater agreement was evaluated on a random sample and exceeded 90%, indicating satisfactory consistency. Any discrepancies were resolved through discussion and, when necessary, adjudication by a third reviewer.

### 2.6 Data extraction

The data extracted comprised the following elements: authors, year of publication, country of study, study design, sample size, participant characteristics, instruments employed to measure neuroticism, instruments used to evaluate self-management, reported levels of neuroticism, reported levels of self-management behaviors, and principal findings. All extracted data were systematically collated using a standardized data extraction form to ensure consistency and to facilitate subsequent analysis and tabular presentation.

### 2.7 Data analysis

Data analysis integrated traditional descriptive synthesis with machine learning-assisted text mining to explore thematic patterns and conceptual linkages across the included literature. The workflow was designed to enhance transparency, reproducibility, and interpretability.

#### 2.7.1 Text preprocessing

All textual data were processed through a standardized natural language processing (NLP) pipeline that included tokenization, lemmatization, and stop-word removal. Sentences containing reference lists, numeric identifiers (DOIs), or author information were excluded to ensure semantic clarity. Tokens shorter than three characters or containing numerals were filtered out. Common academic stop-words such as et al. and doi were removed. The final corpus comprised 1,933 valid sentences and 23,738 effective tokens.

#### 2.7.2 Topic modeling with LDA

Latent Dirichlet Allocation (LDA) was used to uncover latent thematic structures within the corpus. Candidate topic numbers (K = 2–10) were compared using coherence scores, and the optimal model (K = 5, coherence = 0.4407) was selected. Vocabulary thresholds were applied (minimum frequency ≥ 2, maximum document coverage ≤ 80%), resulting in 1,700 retained tokens. LDA was implemented using the gensim package (v4.3) in Python 3.10, with default hyperparameters (*α* = 0.1, *β* = 0.01). Each topic was manually reviewed and labeled according to its dominant keywords and representative sentences.

#### 2.7.3 Semantic embedding and clustering with BERT

To capture deeper contextual semantics, the bert-base-uncased pretrained model from the transformers library (v4.30) was applied to encode each sentence into 768-dimensional embeddings. K-means clustering was used to identify latent semantic clusters, with the optimal number (K = 3) determined via the elbow method and silhouette coefficient (peak = 0.0805). Dimensionality reduction for visualization was performed using principal component analysis (PCA). BERT was selected for its capacity to represent contextual and syntactic nuance beyond surface-level word co-occurrence.

#### 2.7.4 Keyword co-occurrence network

A domain-specific co-occurrence network was constructed to visualize relationships among key terms related to neuroticism, self-management, dietary control, medication adherence, blood glucose monitoring, and physical activity. TF-IDF weighting and cosine similarity were used to quantify the connection strengths between keywords. Community detection via modularity optimization was applied to identify conceptual clusters and central nodes.

#### 2.7.5 Software and version control

All analyses were performed in Python 3.10 using the following packages: NLTK 3.8 for text preprocessing, gensim 4.3 for topic modeling, transformers 4.30 for BERT encoding, scikit-learn 1.3 for clustering and PCA, and NetworkX 3.1 for network visualization. All scripts and parameters were archived for reproducibility.

#### 2.7.6 Quality control and manual validation

Two independent reviewers examined the outputs of LDA and BERT analyses to ensure consistency between automatic and manual interpretations. Discrepancies were resolved through discussion and consensus, and, where necessary, models were iteratively refined. Manual inspection of representative sentences was performed for each theme to confirm semantic validity and minimize algorithmic bias.

#### 2.7.7 Rationale for model selection

The choice of LDA and BERT was guided by the scoping review’s goal of mapping thematic breadth rather than quantifying causal relationships. LDA was used to identify probabilistic topic structures based on lexical co-occurrence, providing an interpretable overview of how neuroticism and self-management are discussed across studies. In contrast, BERT embeddings capture contextual semantics, allowing the identification of subtle conceptual proximity beyond surface word patterns. The combination of these two methods offers complementary perspectives—LDA reveals macro-level thematic distributions, while BERT highlights micro-level semantic relationships—thereby enhancing both the depth and transparency of the evidence mapping process.

### 2.8 Methodological considerations and quality appraisal

Following the methodological guidance of PRISMA-ScR and the JBI Manual for Evidence Synthesis, this review did not include a formal quality appraisal or risk-of-bias assessment. As a scoping review, its purpose was to map and describe the research landscape on neuroticism and self-management in type 2 diabetes, rather than to evaluate methodological rigor or synthesize effect sizes. To ensure transparency, descriptive information on study design, sample characteristics, measurement tools, and analytical approaches was summarized in the Results section. This approach aligns with international standards for scoping reviews and maintains consistency between the study’s exploratory aim and methodological framework.

## 3 Results

**Figure 1:**
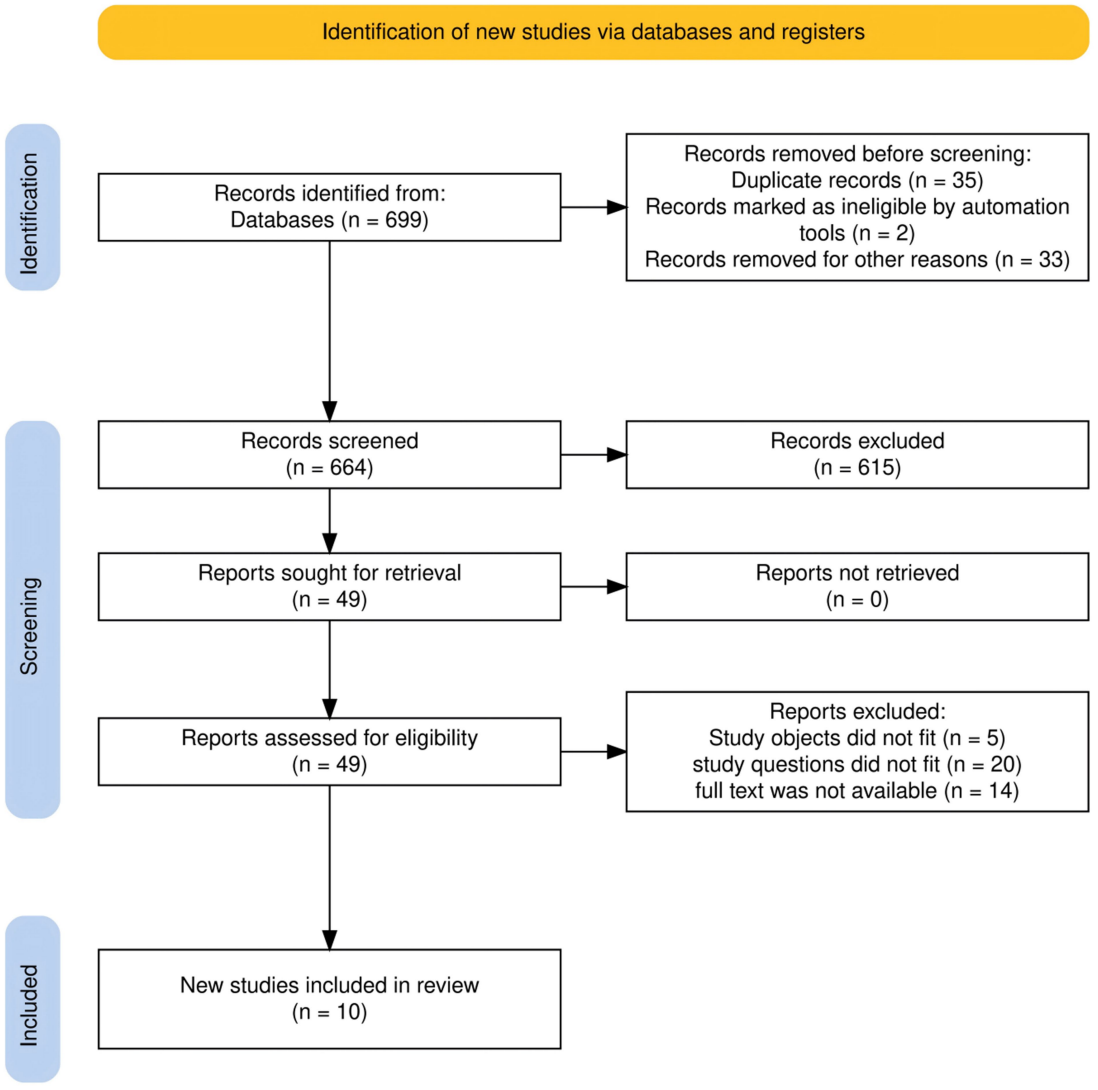
Literature selection flowchart illustrates the identification, screening, eligibility assessment, and inclusion stages in the scoping review on the relationship between neuroticism and self-management behaviors in adults with type 2 diabetes mellitus (Scoping review, global, 2025).

The initial search identified 699 records. After removing duplicates, 664 records remained, of which 10 studies met the inclusion criteria and were ultimately included. The detailed study selection process is shown in Figure 1.

### 3.1 Characteristics of the included studies

A total of 10 studies were included in this scoping review, originating from 6 countries: China (*n* = 3), the United States (*n* = 2), Mexico (*n* = 1), Bosnia and Herzegovina (*n* = 1), Iran (*n* = 2), and Australia (*n* = 1). The included studies comprised eight cross-sectional designs, one prospective cohort, and one longitudinal study. The methodological characteristics, sample features, and measurement tools of each study are summarized in Table 1.

**Table 1:** Key characteristics of studies included in the scoping review of the influence of neuroticism on self-management behaviors in patients with type 2 diabetes (Scoping review, global, 2025).

Author(s)	Year of publication	Country	Study design	Sample size (*n*)	Participant characteristics	Neuroticism assessment tool	Self-management assessment tool	Neuroticism level	Self-management behavior level	Main conclusions
Milicevic et al. (17)	2015	Bosnia and Herzegovina	CS	91	Mean patient age 62.41 years; 47.3% with primary education, 37.4% with secondary education; 75.8% married; 71.4% with poor adherence to regular doctor visits	DS-14; Bortner Scale	No specific self-management assessment tool used; adherence evaluated via frequency of blood glucose monitoring and primary care visits	51.6% of patients were D-type personality and 76.9% were B-type personality	Overall adherence: 67.0% for blood glucose monitoring, 28.6% for doctor visits; among D-type patients: 59.0% for blood glucose monitoring, 34.6% for doctor visits	High neuroticism tendency is associated with poorer medical visit adherence and suboptimal health behaviors in patients with diabetes
Esmaeilinasab et al. (45)	2016	Iran	CS	400	Male: 64%, female: 36%; mean age: 51.2 years; 83.3% married; 40% with a high school education; duration of diabetes: 1–30 years; 71.8% using oral hypoglycemic agents	NEO-PI	No self-management assessment tool was used independently; glycemic control was evaluated through HbA1c	Neuroticism was positively correlated with HbA1c, but the specific scores were not directly reported	The mean HbA1c was 7.0%, overall approaching the optimal control level	Extraversion and conscientiousness: negative correlation with HbA1c; neuroticism and impression management: positive correlation; neurotic defense mechanisms: mild negative correlation with HbA1c.
Novak et al. (18)	2017	United States	CS	234	Mean patient age 57.4 years, mean disease duration ~11 years; spouse mean age 57.4 years; 61.5% first marriage, mean marriage duration 29.4 years; ~75% of patients and 80% of spouses with mild depressive symptoms	BFI	SDSCA	The mean neuroticism score was 2.81 for patients, and 2.68 for spouses	The mean HbA1c was 7.0%, overall approaching the optimal control level	Extraversion and conscientiousness: negative correlation with HbA1c; neuroticism and impression management: positive correlation; neurotic defense mechanisms: mild negative correlation with HbA1c
Lane et al. (46)	2000	United States	LCS	105	Age range 31–82 years, mean 56.9 years; 56% male, 44% female; 87% White, 12% African American; diabetes duration 1–40 years, mean 5 years; 73% using antidiabetic medications	NEO-PI-R	Self-monitoring of blood glucose (SMBG, 7-day average glucose) and HbA1c (reflecting 12–16 weeks of glycemic control)	High neuroticism was linked to poorer glycemic control and more pronounced negative emotions, though specific values were not reported.	Mean SMBG 7.99 mmol/Land HbA1c 7.8%; higher neuroticism was associated with lower SMBG and HbA1c (better glycemic control).	Neuroticism and its facets were associated with better glycemic control, whereas altruism was linked to poorer control; both predicted SMBG/HbA1c over follow-up.
Sanatkar et al. (30)	2020	Australia	LS	99	Mean age 60 years; 55.3% female; mean age at diabetes diagnosis 48.83 years	BFI	SMP-T2D; DDS, PHQ-9, GAD-7	Mean neuroticism score was 3.19, positively correlated with PHQ-9 and GAD-7	Mean scores: healthy eating 3.44, exercise 3.46, SMBG 3.71; low neuroticism associated with decreased depression, anxiety, and diabetes distress at 12 months	Low neuroticism associated with reduced depression, anxiety, functional impairment, and diabetes distress over 12 months; high neuroticism showed no change
Hazrati-Meimaneh et al. (24)	2020	Iran	CS	495	Mean age 57.59 years; 73.5% female; 96.8% living in urban areas; 80.8% married; 65.9% with less than 12 years of education; 66.1% unemployed; mean diabetes duration 10.29 years	NEO-PI-R	SDSCA; MMAS-8	Mean neuroticism score 2.73; females scored significantly higher (2.84 ± 0.82) than males (2.39 ± 0.81)	Mean medication adherence score 6.36; mean total self-care score 53.50	Neuroticism was negatively correlated with medication adherence and self-care, with stronger negative effects observed in females
Huang et al. (19)	2021	China	CS	483	60.0% male, mean age 60.22 years; 64.6% with high school education or below; 87.0% married; 68.3% unemployed; 44.3% with diabetes duration 5–14 years; 50.3% using only antidiabetic medication	EPQ-RS	MMAS-8	The median neuroticism score was 2.0; adherent patients had a median score of 1.0, while non-adherent patients had a median score of 2.0	The mean HbA1c was 7.0%, overall approaching the optimal control level	Both social support and self-efficacy positively influenced medication adherence, whereas neuroticism negatively affected adherence through these factors and their chain-mediated effects
Lee et al. (47)	2021	Taiwan, China	CS	214	Mean age 55.6 years; 57% female; 87% married; 40.7% ≥ university education; mean diabetes duration 8.9 years; 79.4% with comorbidities	QB4	No specific self-management assessment tool was used; management was indirectly evaluated via HbA1c (≥7% indicating hyperglycemia) and fasting blood glucose	The median neuroticism score was 2.0; the high HbA1c group (≥7%) also had a median score of 2.0	78.5% had high HbA1c; patients in precontemplation/contemplation exercise stages showed higher levels	Neuroticism independently predicted high HbA1c; affected patients had poorer glycemic and lipid profiles, especially those in precontemplation/contemplation exercise stages
Mendoza-Catalán et al. (48)	2022	Mexico	CS	197	74.6% female, 25.4% male; mean age 53.1 years; mean years of education 5.7; mean diabetes duration 9 years; mean BMI 27.5 kg/m^2^, 59.8% with high body fat	BFI	SDSCA	Mean neuroticism score 23.4	Mean total self-management score was 28.0, with the lowest score for blood glucose monitoring (1.6 ± 1.2) and the highest for diet (15.8 ± 6.6)	Conscientiousness and openness were positively correlated with self-management, while neuroticism was negatively correlated
Deng L et al. (20)	2023	China	CS	240	59.2% female; 98.3% married; 40.8% aged 50–60 years; 34.6% with junior high education; 44.2% farmers; 38.8% with household monthly income 2000–4,000 RMB; 78.3% living with spouse; 39.6% with diabetes duration ≥10 years	CBF-PI-S	DSMB	Mean neuroticism score 27.53, with an average item score of 3.441	Mean total self-management score 36.98, with poorer performance in blood glucose monitoring	High conscientiousness and low neuroticism were linked to better self-management, with temporal decision-making partially mediating these relationships

CS, cross-sectional study; LCS, longitudinal cohort study; LS, longitudinal study; DS-14, Type D Personality Scale-14; Bortner Scale, Bortner Type A Behavior Pattern Scale; NEO-PI, NEO Personality Inventory; HbA1c, glycated hemoglobin A1c; BFI, Big Five Inventory; SDSCA, Summary of Diabetes Self-Care Activities; NEO-PI-R, NEO Personality Inventory—Revised; SMBG, Self-Monitoring of Blood Glucose; SMP-T2D, Self-Management Program for Type 2 Diabetes; DDS, Diabetes Distress Scale; PHQ-9, Patient Health Questionnaire-9; GAD-7, Generalized Anxiety Disorder-7; MMAS-8, 8-Item Morisky Medication Adherence Scale; EPQ-RS, Eysenck Personality Questionnaire—Revised Short Scale; QB4, Questionnaire Battery 4; BMI, Body Mass Index; CBF-PI-S, Chinese Big Five Personality Inventory—Short Version; DSMB, Diabetes Self-Management Behavior Scale.

### 3.2 Keyword frequency analysis and identification of core concepts

#### 3.2.1 Distribution patterns of high-frequency terms

**Figure 2:**
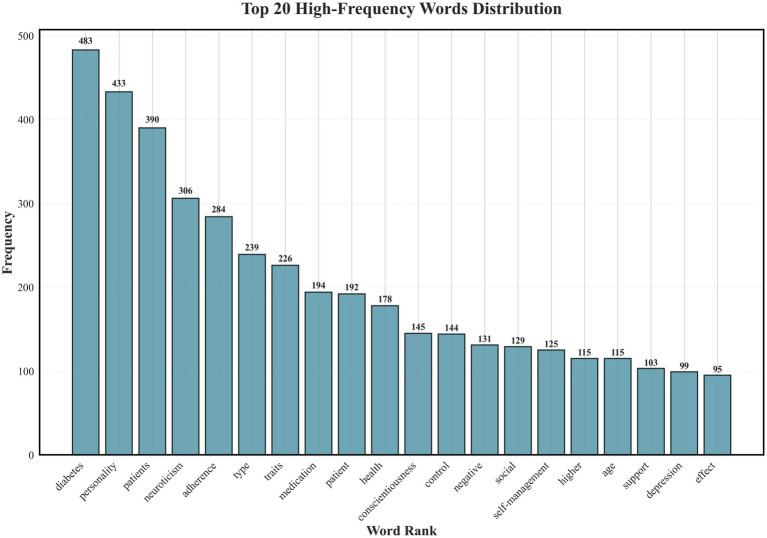
High-frequency keyword analysis depicts core concepts discussed in studies on the relationship between neuroticism and self-management behaviors in adults with type 2 diabetes mellitus (Scoping review, global, 2025).

High-frequency keyword analysis showed that “diabetes” (*n* = 483), “personality traits” (*n* = 433), and “patient population” (*n* = 390) were the most frequently discussed terms, reflecting the general research focus and population scope. The term “neuroticism” appeared 306 times, highlighting its central thematic role. Among self-management–related terms, “adherence” (*n* = 284), “medication” (*n* = 194), and “self-management” (*n* = 125) were common. Psychosocial factors such as “social support” and “depression” were frequently co-mentioned, suggesting that emotional and social dimensions are prominent in self-management discourse. These co-occurrence frequencies describe discussion prominence rather than empirical association. Results are visualized in Figure 2.

#### 3.2.2 Co-mention patterns of major high-frequency terms

**Figure 3:**
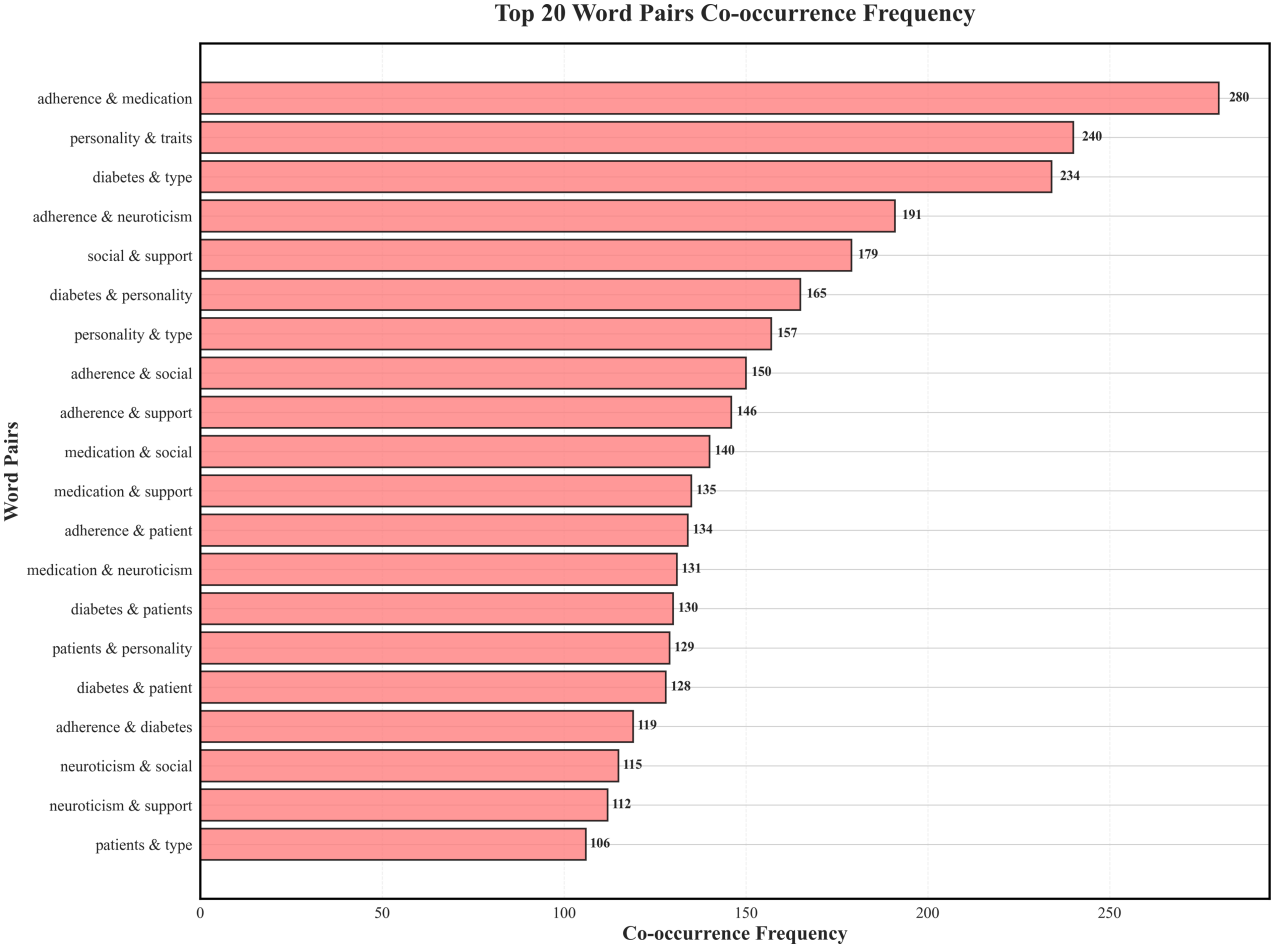
Co-mention analysis of major high-frequency terms illustrates core thematic linkages in studies on neuroticism and self-management behaviors in adults with type 2 diabetes mellitus (Scoping review, global, 2025).

Co-mention analysis indicated that the most frequently co-mentioned pairs were “adherence–medication” (280 occurrences), “personality–traits” (240), and “diabetes–type” (234). Other notable co-mentions included “social–support” (179), “diabetes–personality” (165), “personality–type” (157), “adherence–social” (150), and “adherence–support” (146), suggesting a prominent thematic linkage between social factors and adherence. Neuroticism-related co-mentions were also salient—“medication–neuroticism” (131), “neuroticism–social” (115), and “neuroticism–support” (112)—highlighting the discursive proximity of neuroticism to medication and social-support themes across the literature. These frequencies characterize patterns of discussion rather than statistical or causal relationships. Detailed results are shown in Figure 3.

### 3.3 Thematic structure analysis

#### 3.3.1 Optimal number of topics

**Figure 4:**
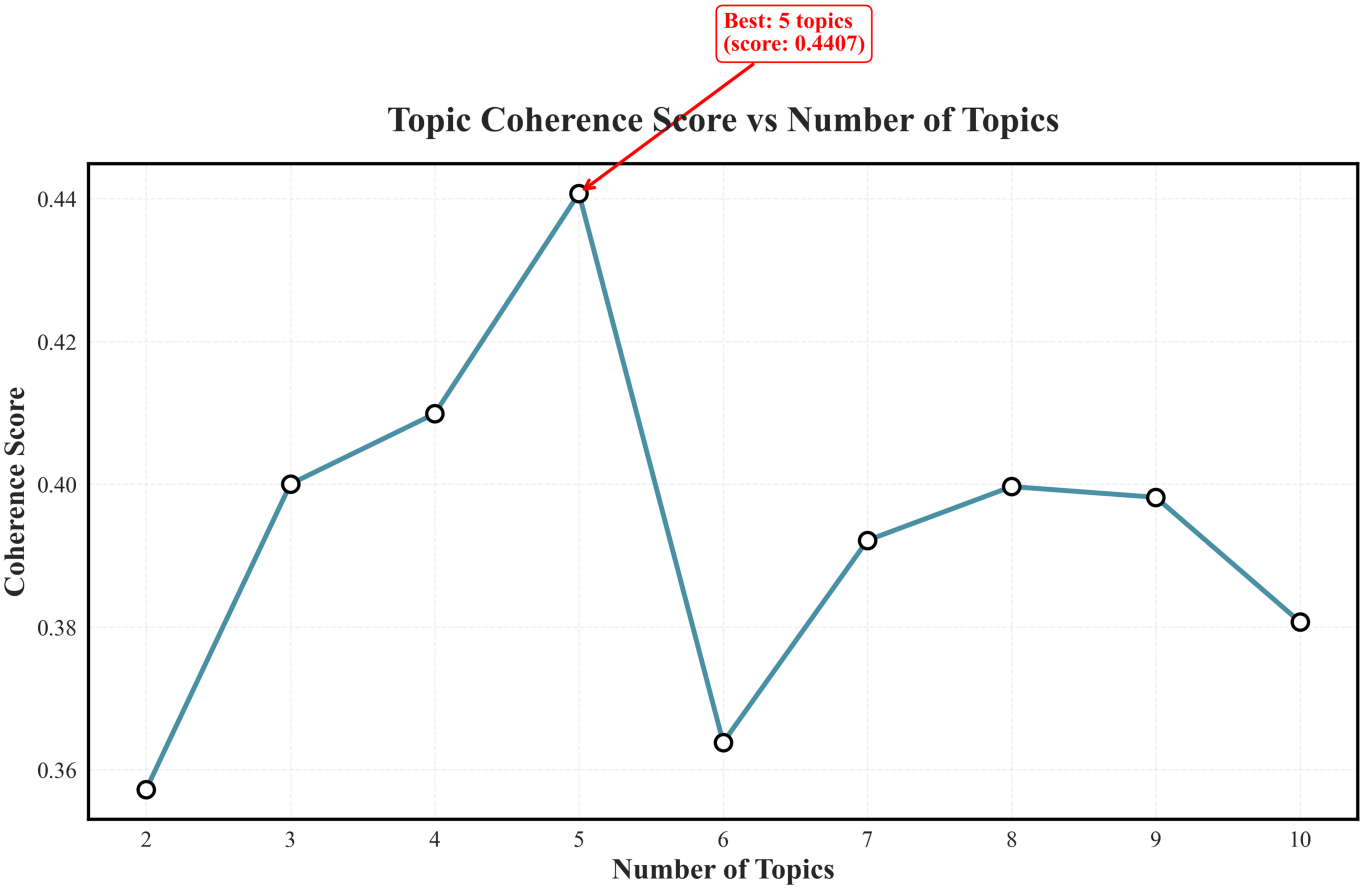
Optimization of topic number shows the five-topic solution representing major discussion themes in the scoping review on neuroticism and self-management (Scoping review, global, 2025).

The optimization of topic coherence in LDA modeling indicated that a five-topic solution (K = 5) achieved the best coherence score (0.4407). This configuration provided balanced interpretability and model stability, capturing conceptual diversity within the literature on neuroticism and diabetes self-management. Thematic structures are illustrated in Figure 4.

#### 3.3.2 Visualization of thematic terms

**Figure 5:**
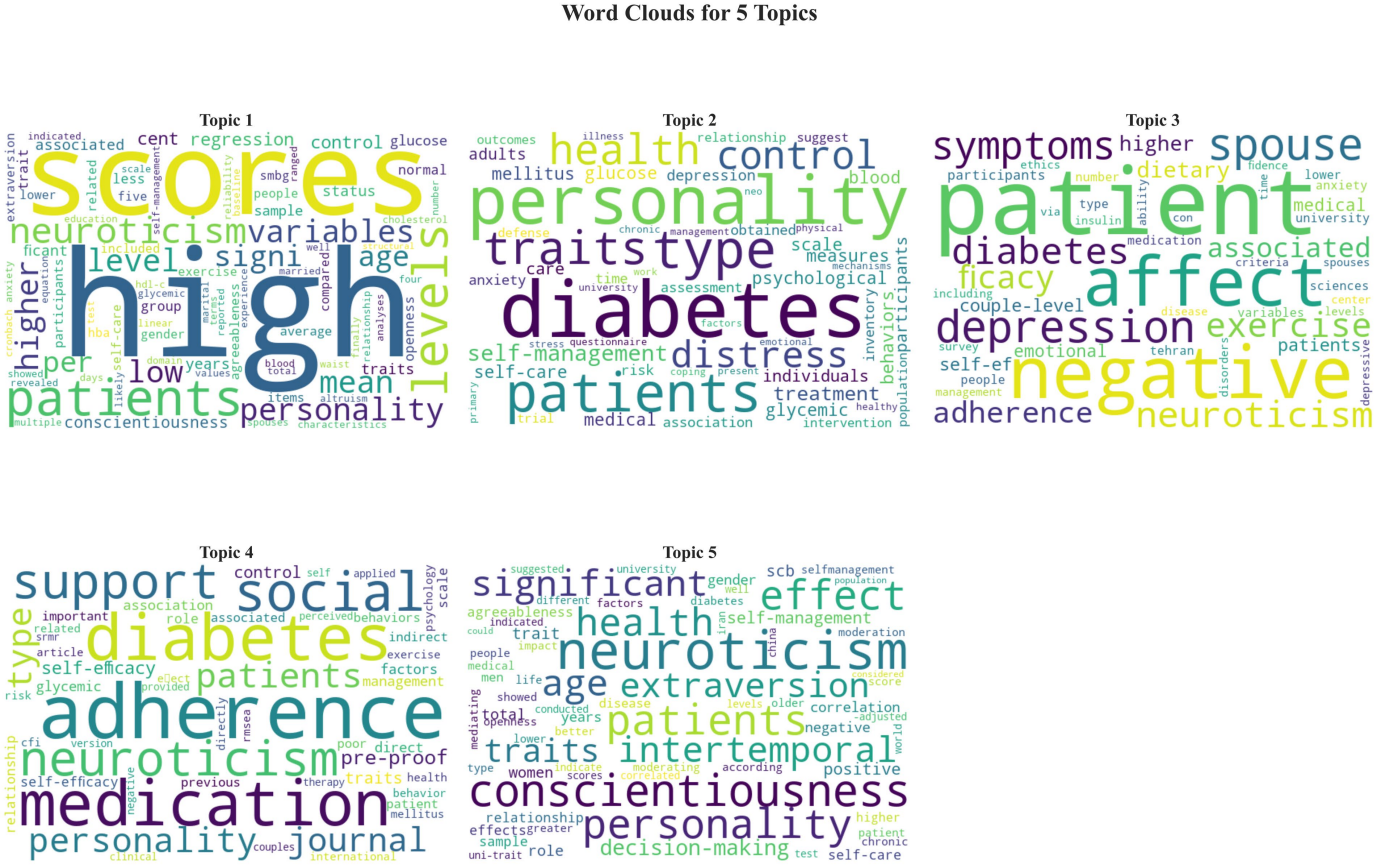
Visualization of thematic terms depicts topic clusters discussed in studies on neuroticism and self-management behaviors among adults with type 2 diabetes mellitus (Scoping review, global, 2025).

The visualization of thematic terms produced five discernible topic clusters, each representing a distinct domain of discussion within the literature. Topic 1 was primarily associated with statistical terminology, including words such as “level,” “score,” “variable,” and “measurement,” reflecting methodological discourse. Topic 2 focused on foundational personality constructs, characterized by the frequent use of “personality traits,” “diabetes,” “type,” and “characteristics.” Topic 3 encompassed emotional and cognitive aspects, featuring terms such as “patients,” “negative,” “emotions,” and “stress.” Topic 4 concentrated on behavioral adherence and self-management, with commonly co-mentioned terms like “adherence,” “diet,” “exercise,” and “blood glucose monitoring.” Topic 5 reflected comparative personality discussions, where “neuroticism,” “conscientiousness,” and “extraversion” frequently appeared together. Collectively, these clusters depict how discussions of neuroticism are distributed across methodological, emotional, and behavioral domains in the existing literature. The visualization of these thematic structures is presented in Figure 5.

#### 3.3.3 Detailed distribution of thematic term weights

**Figure 6:**
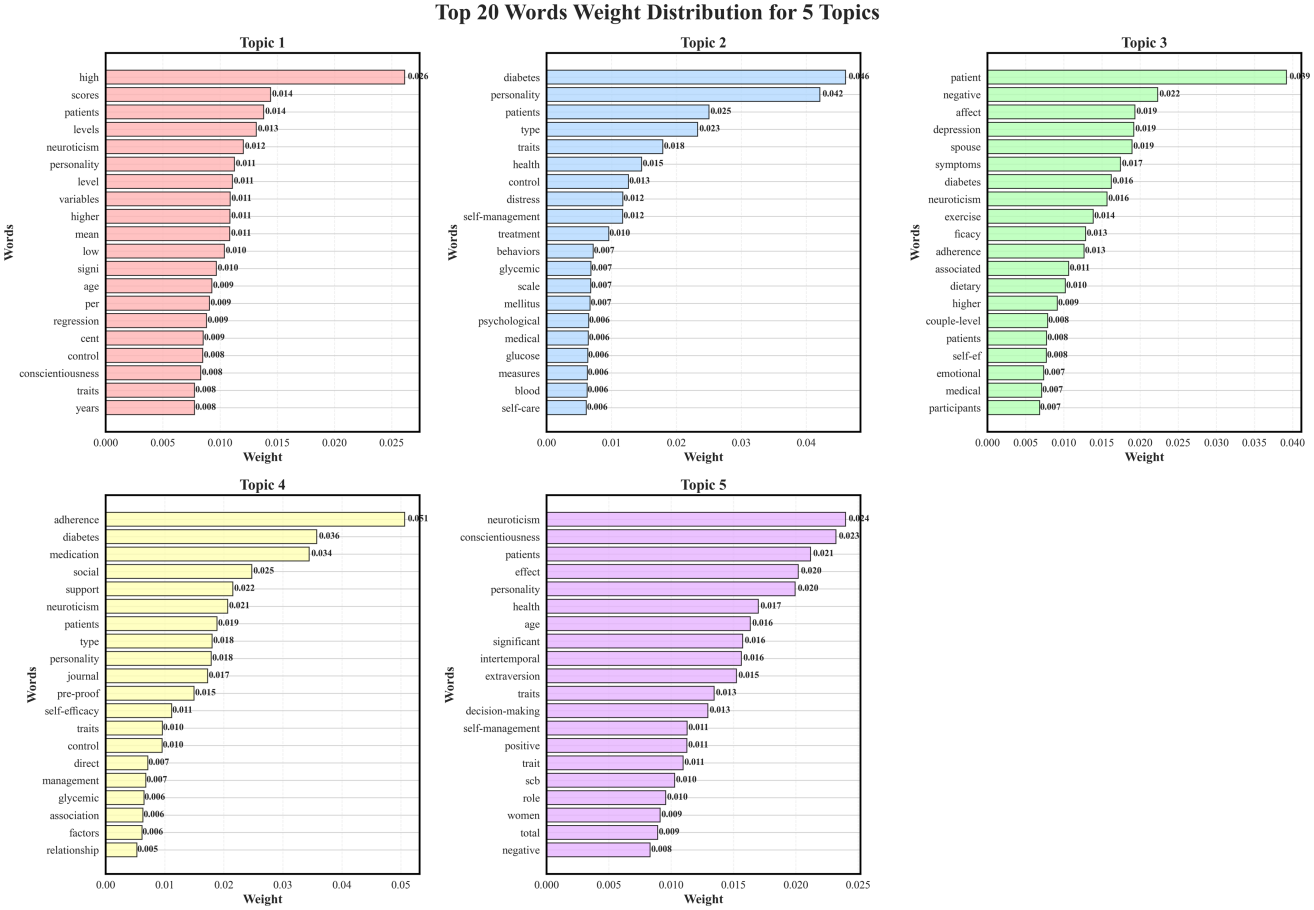
Distribution of thematic term weights depicts the hierarchical arrangement of topics in studies on neuroticism and self-management behaviors among adults with type 2 diabetes mellitus (Scoping review, global, 2025).

The analysis of term weights across topics revealed a hierarchical distribution of thematic content within the literature. In Topic 4, the term “adherence” (0.0506) had the highest frequency weight, indicating that adherence-related terminology appeared more often within behavioral management discussions. Topic 2 displayed comparatively higher weights for “diabetes” (0.0461) and “personality traits” (0.0421), which were recurrently used in conceptual and theoretical contexts. Topic 3 included “patients” (0.0392), “negative” (0.0223), and “emotions” (0.0193), representing terms that occurred more frequently in references to emotional and cognitive aspects. In Topic 5, “neuroticism” (0.0240) and “conscientiousness” (0.0232) showed similar frequency weights, reflecting their repeated co-mention in discussions of comparative personality characteristics. Collectively, these distributions describe the relative prominence of thematic terms across research domains, outlining the structural organization of topic-specific discourse rather than inferential relationships. Detailed results are presented in Figure 6.

### 3.4 Keyword co-occurrence network analysis

**Figure 7:**
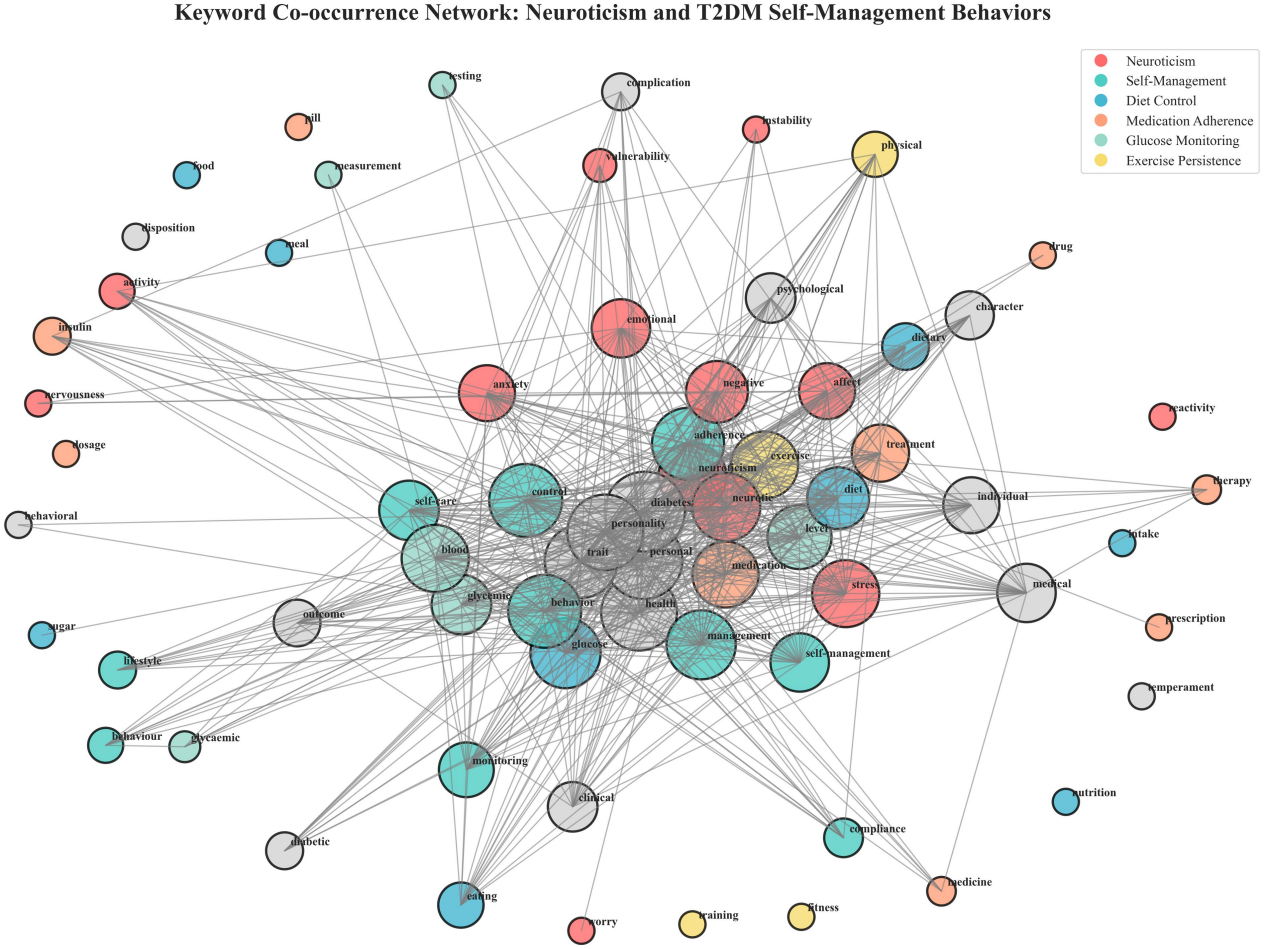
Keyword co-occurrence network illustrates the semantic relationships between neuroticism, emotional constructs, and self-management discussions in adults with type 2 diabetes mellitus (Scoping review, global, 2025).

The keyword co-occurrence network showed that the “neuroticism” cluster connected emotional terms (“stress,” “anxiety,” “negative affect”) with behavioral terms (“adherence,” “self-management”). This highlights neuroticism’s role as a semantic bridge linking emotional reactivity and behavioral regulation within research discourse. The visualization is shown in Figure 7.

### 3.5 Thematic mapping of neuroticism in self-management discussions

#### 3.5.1 Co-occurrence patterns of neuroticism and self-management behaviors

**Figure 8:**
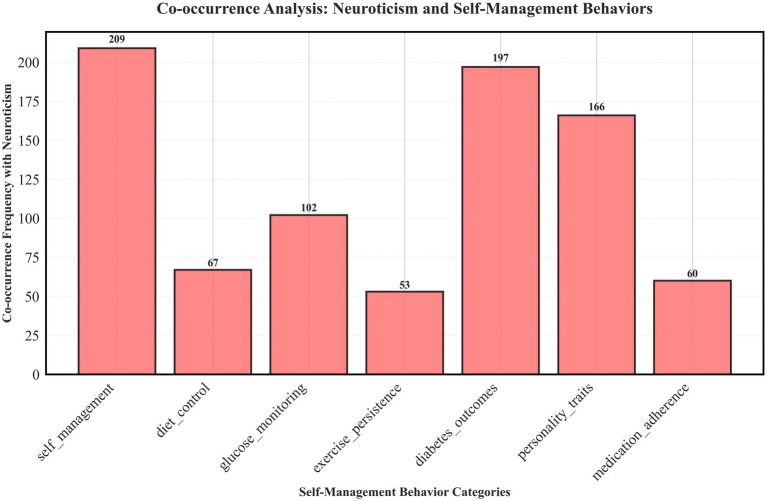
Co-mention patterns between neuroticism and self-management themes across studies (Scoping review, global, 2025).

Across the literature, neuroticism was most frequently co-mentioned with general self-management behaviors (209 times), followed by blood-glucose monitoring (102), dietary control (67), medication adherence (60), and exercise maintenance (53). These frequencies indicate differences in research emphasis rather than measured associations. Neuroticism was also often co-mentioned with “diabetes outcomes” (197) and “conscientiousness” (166), reflecting its comparative role in personality research. Figure 8 illustrates these thematic connections.

#### 3.5.2 Conceptual knowledge framework

**Figure 9:**
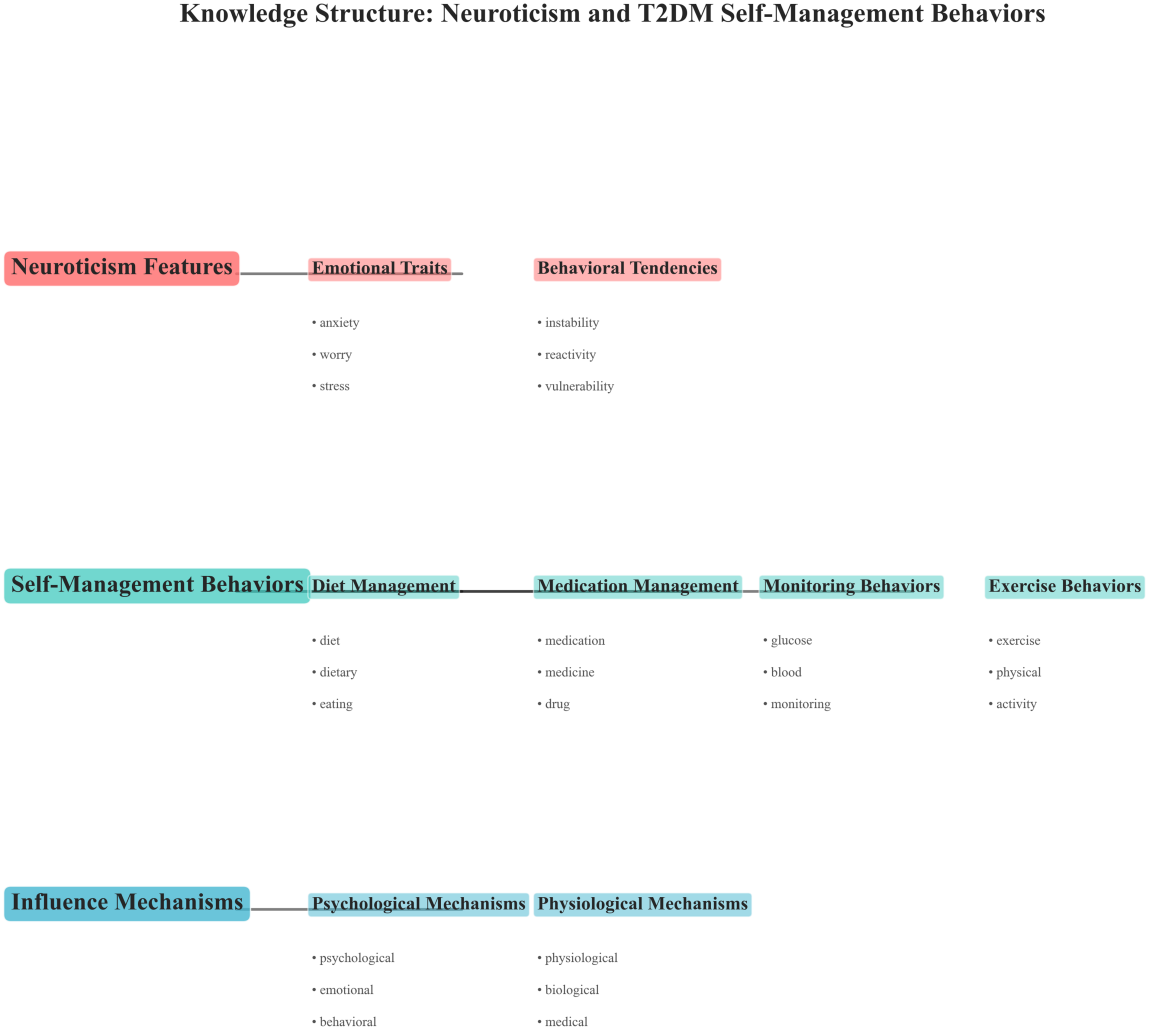
Conceptual framework visualizes thematic linkages between neuroticism and self-management discussions in adults with type 2 diabetes mellitus (Scoping review, global, 2025).

Based on LDA and BERT analyses, a conceptual framework (Figure 9) was constructed to visualize thematic linkages between neuroticism and self-management-related constructs. The framework was automatically derived from topic co-mention frequencies and semantic similarities. Arrows indicate semantic directionality within the discussions rather than causal or statistical effects. The model demonstrates how neuroticism connects with behavioral domains (diet, medication, exercise, monitoring) and psychological correlates (stress, emotion, coping), reflecting the structural organization of existing research.

## 4 Discussion

### 4.1 Patterns of discussion on neuroticism in relation to self-management behaviors among patients with type 2 diabetes

Multi-level text-mining analyses indicated that neuroticism was the most frequently discussed personality construct in the literature addressing self-management behaviors among patients with type 2 diabetes mellitus (T2DM). Across the studies, neuroticism was most often discussed in conjunction with blood glucose monitoring, followed by dietary control, medication adherence, and physical activity (17). Rather than suggesting stronger statistical associations, this distribution reflects the frequency and prominence of neuroticism-related discourse in these behavioral domains. Within the reviewed literature, neuroticism was frequently co-mentioned with psychosocial concepts such as anxiety, stress sensitivity, social support, and self-efficacy (18–20). These co-occurrences suggest that neuroticism has been positioned as a psychological interpretive construct—a framework through which emotional and behavioral variability in self-management is discussed. In this context, neuroticism serves as a recurring theme linking emotional instability to self-regulatory challenges in diabetes care, illustrating how emotional reactivity and perceived stress are discussed in relation to patient engagement and self-management. Importantly, several studies contextualized neuroticism by comparing it with other personality dimensions, most notably conscientiousness, agreeableness, and extraversion (21–23). These comparative discussions framed conscientiousness as representing behavioral discipline and goal orientation, while neuroticism was described as reflecting emotional vulnerability and response sensitivity. Such comparisons help delineate the conceptual boundaries of neuroticism, highlighting its distinctive theoretical position within the personality–behavior discourse. Together, these thematic patterns suggest that neuroticism is not only a frequently discussed topic but also serves as a contrastive reference point for understanding the broader personality dynamics involved in diabetes self-management.

### 4.2 Discussion patterns of neuroticism across domains of self-management behaviors in type 2 diabetes

#### 4.2.1 Neuroticism and dietary control

Across the included studies, neuroticism was frequently discussed in the context of dietary management, ranking second in prominence among the four domains of self-management behaviors. The literature generally describes individuals with higher neuroticism as exhibiting complex psychological responses when making dietary decisions. On the one hand, such individuals were often portrayed as having heightened health awareness and increased attention to dietary regulation; on the other hand, emotional instability and sensitivity to stress were frequently noted in association with behaviors such as emotional eating, which may undermine consistent dietary management (18, 20, 24). Overall, these findings in the literature indicate that dietary regulation is commonly discussed through a psychological and emotional interpretive framework, in which neuroticism functions as a descriptive construct linked to emotional reactivity, rather than a directly measured determinant of behavior (25–28).

#### 4.2.2 Neuroticism and medication adherence

In the reviewed evidence, medication adherence appeared less frequently in discussions of neuroticism compared with other self-management behaviors. Neuroticism was typically described as a contextual factor rather than a principal determinant of medication adherence. The literature emphasized that adherence behaviors are largely discussed in relation to external contextual variables, such as healthcare professional guidance, medication side effects, economic conditions, and family support (19, 24, 29). Within these discussions, neuroticism was occasionally co-mentioned with psychosocial constructs such as trust, self-efficacy, and treatment perception, suggesting its role as an interpretive element in understanding variability in adherence behavior, rather than as an independent causal variable.

#### 4.2.3 Neuroticism and blood glucose monitoring

Blood glucose monitoring was the self-management behavior most prominently discussed in connection with neuroticism across the included studies. The literature often framed this relationship within the context of health vigilance, risk perception, and monitoring frequency (19, 24, 30). Individuals characterized by higher neuroticism were frequently described as showing increased attentiveness to glucose fluctuations and greater sensitivity to health uncertainty, which was discussed as part of a broader psychological sensitivity framework. The operational simplicity and quantifiable feedback nature of glucose testing were also mentioned as potential reasons why monitoring behaviors are more extensively discussed in connection with neuroticism (31–33). Collectively, these patterns in the literature highlight that blood glucose monitoring is the domain where discussions of neuroticism appear most prominently, often in association with emotional vigilance and reassurance-seeking tendencies, rather than through empirical causal examination.

#### 4.2.4 Neuroticism and exercise maintenance

Among the four domains of self-management, physical activity and exercise appeared least frequently in discussions involving neuroticism. When addressed, the literature often related neuroticism to motivation, self-regulation, and emotional barriers, describing it as a psychological attribute that may contribute to difficulties in sustaining exercise behaviors (18, 20). However, these interpretations were largely descriptive, focusing on theoretical or observational discussions rather than empirical confirmation. The relative scarcity of this theme in the reviewed literature may reflect the methodological challenges associated with assessing long-term exercise adherence within personality-related studies. As such, neuroticism was discussed primarily within the broader context of emotional stability and behavioral consistency, rather than as a quantitatively assessed predictor (34–36).

### 4.3 Thematic reflections and practical relevance

Within the reviewed literature, discussions linking neuroticism to self-management behaviors among adults with type 2 diabetes have largely centered on psychological adaptation, emotional regulation, and contextual support systems. Neuroticism is frequently discussed in relation to emotional instability, stress reactivity, and self-efficacy, particularly within the domains of dietary regulation, medication use, blood glucose monitoring, and physical activity (37–39). Rather than presenting neuroticism as a direct determinant of behavior, studies commonly frame it as a personality-based context shaping how patients perceive, interpret, and maintain self-management behaviors. At the public health level, this discourse reflects a growing recognition of personality-informed strategies in chronic disease prevention and management. Several studies emphasized the role of social and community resources—such as communication with healthcare providers, family engagement, and peer support—in moderating the challenges associated with neurotic tendencies (40–42). These themes align with the broader goals of public health practice, which emphasize psychosocial determinants of health, health literacy, and individualized behavioral interventions. From a systems perspective, integrating personality traits into chronic disease management frameworks may enhance population-level interventions by promoting patient-centered education, digital health engagement, and community-based support initiatives (43, 44). Collectively, the reviewed discussions suggest an emerging interdisciplinary convergence between psychological science and public health, highlighting the importance of addressing personality diversity to support equitable and sustainable diabetes self-management.

### 4.4 Added value of machine learning–assisted text mining

In this scoping review, the integration of machine learning–assisted text mining offered methodological advantages that complemented conventional manual synthesis. Techniques such as natural language processing (NLP), latent Dirichlet allocation (LDA) topic modeling, and BERT-based semantic clustering enabled the identification of latent thematic structures and semantic linkages that might not have been detected through traditional narrative synthesis. The computational analysis revealed implicit connections between neuroticism, psychosocial constructs, and specific self-management behaviors—such as the frequent co-mentioning of neuroticism with blood glucose monitoring and social support—that were not consistently highlighted across studies. Moreover, the convergence between algorithmic outputs and manual thematic coding enhanced the transparency and reproducibility of the review process by reducing subjective bias. Although the number of included studies was limited (*n* = 10), the application of machine learning was methodologically justified, as it facilitated structured visualization of thematic density and relational patterns within a heterogeneous evidence base. Rather than replacing traditional qualitative synthesis, machine learning served as an augmentative tool, providing a data-driven mapping of conceptual relationships and highlighting underexplored intersections within the literature. This approach illustrates how hybrid human–machine methodologies can enrich scoping reviews by systematically organizing dispersed evidence and supporting more nuanced thematic interpretation.

### 4.5 Limitations

This review was conducted as a scoping review and therefore did not aim to quantify effect sizes or perform subgroup comparisons. Its purpose was to systematically map and describe how neuroticism has been examined in relation to self-management behaviors among adults with type 2 diabetes. The observed variations in research design, population characteristics, and measurement instruments reflect the conceptual and methodological diversity within this field rather than statistical heterogeneity. In accordance with the PRISMA-ScR guidelines, such variation is inherent to scoping reviews, which emphasize the breadth and characteristics of existing evidence. These variations may nonetheless influence the consistency and interpretability of findings, and the results should therefore be viewed as a descriptive mapping of the research landscape rather than a synthesis of comparable quantitative evidence.

## 5 Conclusion

This scoping review summarized how neuroticism is discussed in relation to self-management behaviors among adults with type 2 diabetes mellitus (T2DM). The reviewed literature reflects diverse thematic patterns across dietary regulation, medication use, blood glucose monitoring, and physical activity, emphasizing the psychological and contextual dimensions of self-management. The integration of machine learning–assisted text mining enhanced thematic identification and provided structured insights into research trends. Future research should expand the scope and methodological diversity of evidence to further refine personality-informed approaches in chronic disease management.

## Data Availability

The original contributions presented in the study are included in the article/Supplementary material, further inquiries can be directed to the corresponding author.

## Footnotes

^1^ https://doi.org/10.17605/OSF.IO/54NJD
