# Supplementary material for: Patterns of discussion on neuroticism and self-management behaviors in type 2 diabetes: a scoping review using machine learning-assisted text mining
Source: Front Public Health. 2025 Nov 4;13:1708967. doi: 10.3389/fpubh.2025.1708967 (PMC12623199; doi:10.3389/fpubh.2025.1708967)
Supplement: Supplementary file 1 [file Supplementary_file_1.docx]

Supplementary Material S1

Database: PubMed

Date of search: September 8, 2025

Search conducted according to PRISMA-ScR guidelines

(("Neuroticism"[Mesh] OR neuroticism[Title/Abstract] OR "personality traits"[Mesh] OR "personality trait*"[Title/Abstract] OR "five-factor model"[Title/Abstract] OR "big five"[Title/Abstract] OR "emotional instability"[Title/Abstract]) AND ("Diabetes Mellitus, Type 2"[Mesh] OR "type 2 diabetes"[Title/Abstract] OR T2DM[Title/Abstract] OR "adult-onset diabetes"[Title/Abstract]) AND ("Self-Management"[Mesh] OR "self management"[Title/Abstract] OR "self-care"[Title/Abstract] OR "self care"[Title/Abstract] OR "disease management"[Title/Abstract] OR adherence[Title/Abstract] OR compliance[Title/Abstract] OR "blood glucose monitoring"[Title/Abstract] OR "medication adherence"[Title/Abstract] OR "dietary control"[Title/Abstract] OR "physical activity"[Title/Abstract] OR exercise[Title/Abstract])) NOT ("animal"[MeSH Terms] NOT "human"[MeSH Terms])

Search fields used: [Title/Abstract] and MeSH Terms

Time frame: From database inception to September 8, 2025

Other databases searched: Scopus, Web of Science, Embase, CINAHL, PsycINFO, and Cochrane Library.

Additional step: Manual screening of reference lists of all included studies and relevant reviews.
